# Metabolic and Endocrine Consequences of Bariatric Surgery

**DOI:** 10.3389/fendo.2019.00626

**Published:** 2019-09-19

**Authors:** Isabel Cornejo-Pareja, Mercedes Clemente-Postigo, Francisco J. Tinahones

**Affiliations:** ^1^Unidad de Gestión Clínica Endocrinología y Nutrición, Instituto de Investigación Biomédica de Málaga—IBIMA, Hospital Universitario Virgen de la Victoria, Universidad de Málaga, Málaga, Spain; ^2^Centro de Investigación Biomédica en Red (CIBER) Fisiopatología de la Obesidad y Nutrición (CIBEROBN), Instituto de Salud Carlos III (ISCIII), Málaga, Spain

**Keywords:** bariatric surgery, somatotropic axis, corticotropic axis, gonadal axis, bone metabolism

## Abstract

Obesity is one of the most serious worldwide epidemics of the twenty-first century according to the World Health Organization. Frequently associated with a number of comorbidities, obesity threatens and compromises individual health and quality of life. Bariatric surgery (BS) has been demonstrated to be an effective treatment to achieve not only sustained weight loss but also significant metabolic improvement that goes beyond mere weight loss. The beneficial effects of BS on metabolic traits are so widely recognized that some authors have proposed BS as metabolic surgery that could be prescribed even for moderate obesity. However, most of the BS procedures imply malabsorption and/or gastric acid reduction which lead to nutrient deficiency and, consequently, further complications could be developed in the long term. In fact, BS not only affects metabolic homeostasis but also has pronounced effects on endocrine systems other than those exclusively involved in metabolic function. The somatotropic, corticotropic, and gonadal axes as well as bone health have also been shown to be affected by the various BS procedures. Accordingly, further consequences and complications of BS in the long term in systems other than metabolic system need to be addressed in large cohorts, taking into account each bariatric procedure before making generalized recommendations for BS. In this review, current data regarding these issues are summarized, paying special attention to the somatotropic, corticotropic, gonadal axes, and bone post-operative health.

## Introduction

Obesity is a chronic, progressive, and multifactorial disease involving genetic, metabolic, psychological, and endocrinology-related factors, among others. Obesity-associated comorbidities are numerous and are also related to higher mortality. Obesity is a risk factor for a number of other chronic illnesses related to metabolic syndrome including type 2 diabetes mellitus (T2DM), high blood pressure, dyslipidemia, cardiovascular diseases (CVD), respiratory disorders, joint diseases, psychosocial disorders, and even several types of cancer (including esophagus, colon, pancreas, prostate, and breast) (1).

The World Health Organization (WHO) has defined the term “obesity” as excessive fat accumulation that is harmful to health. Considered the twenty-first century epidemic, obesity is the main health problem in developed countries, reducing life expectancy and presenting a challenge for the global economy. This is a disease with exponential growth, and its rise and prevalence is also affecting the child population. In the last 40 years the proportion of the population that is obese has tripled. In 2016, 39% of adults aged 18 years and over were overweight and about 13% were obese. If nothing changes, the growing trend will continue and these numbers will increase in the coming years (2).

## Bariatric Surgery (BS): A Treatment for Obesity

From the point of view of therapeutics, proper dietary re-education, together with lifestyle modification and physical exercise as well as psychological specialized support for obesity treatment are needed. However, studies such as Look AHEAD showed that weight loss and glycemic control is difficult to maintain in the long term, even with intensive lifestyle intervention (3). Thus, bariatric surgery (BS) is the most effective therapy in the long term for severely obese patients (grade II) with associated metabolic diseases and for morbidly and super morbidly obese patients (grades III-IV). In addition, there is increasing literature that supports the inclusion of BS for the treatment of T2DM and obese patients (1). In this context, a global approach developed by a multidisciplinary unit, which allows a personalized and comprehensive treatment for obese patients as well as the selection of those patients who can be benefit from surgical treatment is extremely relevant.

There are different effective surgical methods employed for obesity treatment and the majority of the most popular bariatric procedures such as Roux-en-Y gastric bypass (RYGB), sleeve gastrectomy (SG), mini-gastric bypass, and biliopancreatic diversion (BPD) are considered safe procedures that are efficient regarding obesity-associated comorbidities and weight loss (4). Nevertheless, there are some differences between bariatric procedures related to the magnitude of changes observed, complication rates or post-surgery morbidities in the short and long term (reintervention rates after surgery, gastroesophageal reflux disease). Due to these differences, an individualized treatment taking into account the characteristics of each patient is necessary (5–8).

After BS, many aspects are modified and imply a reduction in the risk of obesity-associated disorders as well as of all-cause mortality (9–11). Some of the beneficial effects of BS are improved physical function (12), sustained weight loss, reduction of comorbidities such as osteoarthritis and respiratory dysfunction (13), a more favorable metabolic profile, which implies an improvement in quality of life, and the resolution of cardiovascular risk factors (14–19) with lower triglyceride levels and higher HDL-C levels in most patients 1 year after surgery (20, 21).

Although there is extensive literature that claims beneficial health effects with BS, and the post-surgery mortality rate is <1%, these type of procedures are not exempt from long-term complications related to nutritional deficiencies. In general, mainly malabsorptive procedures, e.g., bypass procedures with duodenal exclusion of nutrients and a concomitant decrease in gastric acid, will have a higher risk of micronutrient and macronutrient deficiencies. Consequently, BS patients are at higher risk of developing nutrient deficiency-related disorders such as anemia, certain types of neuropathies or osteoporosis (22, 23).

## Effects of BS on Glycemic Profile and Mechanisms Involved in T2DM Resolution

A number of studies have explored metabolic changes after BS, and the efficiency of BS for the treatment of T2DM is well-established. BS therefore offers a safe and more effective alternative to achieve sustained glycemic control in diabetic obese patients in comparison with intensive medical treatment (24–26), which involves a decrease in chronic micro- and macroangiopathic complications (27).

The various surgical procedures usually employed in BS lead to partial or total T2DM remission of about 34–85.3% depending on the criteria applied to define T2DM remission, and 95% global success in glycemic control (28). When the different bariatric procedures are compared, biliopancreatic diversion (BPD) is the most effective for T2DM treatment, but this is a more complex procedure with higher surgical adverse event rates compared to other bariatric procedures (29). Risstad et al. reported better outcomes related to sustained weight loss, glycemic control and improvement in lipid profile after BPD than RYGB 5 years after surgery (14). Garrido-Sanchez et al. concluded that BPD takes less time to achieve insulin resistance improvement than SG (30).

RYGB is also a highly effective option for T2DM remission. Most patients who underwent RYGB did not require hypoglycemic drugs 1 year post-surgery, and it has been reported 84–90% and 29–50% T2DM remission one and 5 years after RYGB, respectively (31–35). However, T2DM remission rates in the literature vary. Yan et al. reported 56.81% (36.8–90.3%) T2DM remission in their meta-analysis (36), while Chang et al. found 95.15% (88.38–98.8%) T2DM remission (37).

Sleeve gastrectomy has gained popularity and appears to achieve glycemic control rates similar to RYGB but with fewer surgery-associated complications. The randomized controlled trial SMBOSS reported 60% T2DM remission rates after SG compared to 77% remission after RYGB 3 years after surgery (15). The STAMPEDE study compared the efficacy of intensive medical therapy alone or intensive medical therapy plus RYGB or plus SG (5-year follow-up) finding that, 28.6% of patients who underwent RYGB achieved HbA1c ≤6% in comparison with 23.4% of patients who underwent SG and 5.3% of patients who only received intensive medical therapy. The average improvement in HbA1c was 2.1% in surgery cohorts vs. 0.3% in intensive medical treatment cohorts (25). The SLEEVEPASS study showed that the effects of T2DM remitted completely or partially in 49% of patients who underwent SG and in 57% who underwent RYGB with no significant differences between procedures (16). However, it should be mentioned that the comparison between these two procedures based on the current literature is difficult due to study heterogeneity and because some of them are biased. Thus, their findings should be interpreted cautiously and further randomized controlled trials with a careful design are necessary (38).

### Mechanisms for T2DM Remission After BS

Many explanations have been put forth for the metabolic improvements after BS. Early and pronounced improvement in hepatic insulin sensitivity after RYGB, which could be related to caloric restriction in the short-term post-operative period and the reduction in intrahepatic fat, has been reported. Moreover, an improvement in peripheral insulin sensitivity, which occurs later, and its relationship with the sustained weight loss has been described (39, 40).

Nevertheless, it is well-known that the metabolic improvements after BS take place in the days or weeks after surgery even before significant weight loss occurs (21, 41, 42). Therefore, mechanisms other than weight loss that cannot be explained solely by caloric restriction are likely triggered after BS and could account for the beneficial metabolic changes observed. This has promoted the use of the term “metabolic surgery” for this type of procedures (43).

**Figure 1:**
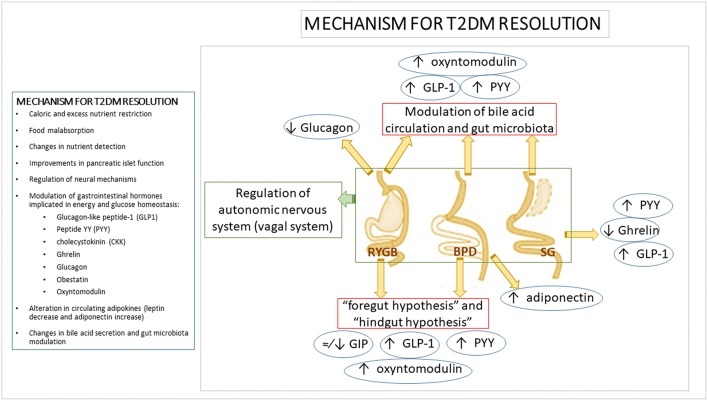
Mechanism for T2DM resolution. Mechanisms and modifications of main gastrointestinal hormones involved in T2DM resolution after bariatric surgery. Several mechanisms have been proposed to explain the metabolic improvement after bariatric surgery. However, due to the fact that each bariatric procedure does not involve the same gastrointestinal tract modifications, it has been suggested that each procedure acts by means of different mechanisms to achieve T2DM resolution, including differential shifts in gastrointestinal hormones levels. It has been proposed that the exclusion of the duodenum and proximal jejunum in bariatric procedures such as RYGB or BPD would inhibit the “anti-incretin” signaling (“foregut hypothesis”). This kind of remodeling would also reduce the time that nutrients take to reach distal jejunum which could imply an early activation of incretin-secreting L-cells in the distal ileum and proximal colon (“hindgut hypothesis”). Incretins such as GLP-1, PYY, or oxyntomodulin improve pancreatic insulin secretion and reduce glucagon release. By contrast, the main gastrointestinal hormonal shift expected after SG is the decrease in the levels of the orexigenic hormone ghrelin due to the removal of the gastric fundus and therefore, of the ghrelin-producing mucosa. However, it has also been described an increase in GLP-1 and PYY levels after SG likely due to a shorter intestinal transit after surgery. Apart from changes in gastrointestinal hormone patterns, alterations in bile acid metabolism, gut microbiota composition, modification in gastrointestinal vagal signaling or changes in adipokines levels described after the different bariatric procedures, could be also involved in the bariatric metabolic improvement and T2DM resolution after surgery. BPD, biliopancreatic diversion; GIP, gastric inhibitory polypeptide; GLP-1, Glucagon-like Peptide 1; PYY, Peptide YY; RYGB, Roux-en-Y gastric bypass; SG, sleeve gastrectomy; T2DM, Type 2 diabetes mellitus.

Several hypotheses have been put forth to explain the early improvement in carbohydrate metabolism after BS, but the precise mechanisms for T2DM resolution are not yet completely understood (Figure 1). Among the factors which could be determining these improvements, in addition to caloric and excess nutrient restriction and the weight loss associated with food malabsorption, are changes in nutrient detection, improvements in pancreatic islet function, modulation of neural mechanisms and the secretion of gastrointestinal hormones implicated in energy and glucose homeostasis (e.g., glucagon-like peptide-1 (GLP1), peptide YY (PYY), cholecystokinin (CKK), ghrelin (44), glucagon (45, 46), obestatin levels (47), alteration in circulating adipokines (leptin decrease and adiponectin increase) (48, 49), bile acid secretion, or gut microbiota modulation (50). Most of the mechanisms proposed to explain the early metabolic improvements that occur independently from weight loss are related to the anatomic remodeling of the gastrointestinal tract, which highlights the relevance of the intestine in carbohydrate metabolism regulation after BS (51, 52).

Several hypotheses have been raised regarding the implication of the gastrointestinal system as a metabolically active organ:

- The “foregut hypothesis” is based on the exclusion of the duodenum and proximal jejunum from the nutrient traffic across the gastrointestinal tract, which may inhibit “anti-incretin” signal production such as gastric inhibitory polypeptide (GIP). The removal of this signaling would favor direct antidiabetic effects with a reduction in insulin resistance (53–55).
- The “hindgut hypothesis” posits that nutrients reach the distal jejunum quickly, ensuring optimal digestion and nutrient absorption as well as metabolic improvement and weight loss through stimulation of L cells in the distal ileum and proximal colon which secrete GLP-1, PYY, oxyntomodulin, and other hormones (56). This response leads to an improvement in insulin secretion by pancreatic β cells and a reduction in glucagon production and hence, to a better post-prandial response to glucose and lower insulin resistance. By contrast, various BS procedures have shown heterogeneous effects on circulating GIP levels, ranging from moderate to unnoticeable and sometimes even leading to a decrease (46, 57–60).
- Other possible mechanisms have been elucidated from the results of SG. Although it would initially be considered a purely restrictive bariatric procedure, evidence has highlighted that its effects cannot be explained by caloric restriction alone. SG also leads to complex hormonal changes such as diminished ghrelin levels, which enhance weight loss and improve post-prandial insulin response together with lower post-prandial glucose levels (61, 62), as well as higher post-prandial GLP-1 and PYY responses induced by a short intestinal transit after surgery (63).
- The modulation of bile acid circulation has been postulated as a feasible mechanism to explain some of the metabolic effects of BS. Bile acids could be involved in both weight loss and the improvement in glycemic control by the stimulation of intestinal L-cell secretion of GLP-1, PYY, and oxyntomodulin which favors insulin response and the feeling of satiety (64, 65). Human and animal studies support the key role of bile acids in glucose metabolism regulation by means of pathways activated by the interaction of bile acid with the nuclear and membrane receptors farnesoid X receptor (FXR) and Takeda G protein-coupled receptor 5 (TGR-5), which are present in a number of tissues and organs such as the gut, liver, pancreas, adipose tissue, and skeletal muscle. By means of these receptors, bile acids lead to an increase in insulin secretion by pancreatic β cells, improvements in glucose tolerance, enhancement of glucose uptake by adipose tissue, decrease in gluconeogenesis, increase in glycogen synthesis and promotion of hepatic glucose catabolism (20, 66–68).
- Another intestine-related effect of BS which could be involved in the BS metabolic effects is the modulation of gut microbiota. Gut microbiota composition has previously been associated with obesity, T2DM and other metabolic diseases (55). Thus, it has been proposed that the modulation of gut microbiota composition by BS might be, at least in part, responsible for the post-surgical metabolic improvements. In agreement with this hypothesis, significant changes in gut microbiota composition have been reported after BS (50), but results from different studies are heterogeneous and no clear conclusions have been drawn (69). RYGB has been associated with an increase in Proteobacteria (70–72), while others described a concomitant decrease in Firmicutes and Bacteroidetes (70, 71). However, other studies found different shifts in gut microbiota profile after BS. Kong et al. showed a decrease in bacteria belonging to Firmicutes phyla at 3 and 6 months after RYGB in obese women (most of them without T2DM) (72) and a recent study found that RYGB lead to a decrease in Bacteroidetes 1 year post-operatively in obese diabetic patients (73). More detailed information regarding changes in specific bacterium groups have been recently reviewed elsewhere, giving an idea of the heterogeneous results (69, 74). Independently of the specific changes in bacterium groups, an increased bacterial diversity (previously related to the healthy status) post-surgery was reported (72, 75). Few studies have analyzed other BS procedures than RYGB regarding their effects on gut microbiota composition (73, 76–78). Similar changes in gut microbiota composition were found after RYGB and vertical banded gastroplasty (VBG) (76). However, recent studies reported differences in post-surgical gut microbiota profile between RYGB and SG (73) and between RYGB and laparoscopic adjustable gastric banding (LAGB) (77). The different design and participant inclusion criteria (gender, presence of T2DM and other obesity comorbidities or follow-up after surgery) of the current studies could explain the discrepancies found. Moreover, the gut microbiota profile after BS can be also dependent on post-surgery analysis time, omeprazole intake and diet (73, 79). Despite firm conclusions regarding the specific intestinal bacterial switch have not yet been drawn, recent studies suggest that gut microbiota composition is related to the success of surgical intervention in terms of excess weight loss or T2DM remission (73, 80). In view of these results, it can be hypothesized that the inter-individual response to BS might be, at least in part, dependent on gut microbiota composition. Furthermore, several studies have reported that BS is related to the modulation of the mechanisms presumably involved in the gut microbiota-host metabolism crosstalk such as LPS translocation and inflammatory markers (41, 70, 81, 82), regulation of GI hormone secretion (77, 83), influence on AT function (72, 84), bile acid metabolism (79), or short-fatty acid (SCFA) production (77). All these evidence point out that there is a relationship between BS consequences and gut microbiota, although further homogeneous studies are necessary in order to get a comprehensive overview of the involvement of gut microbiota in the metabolic improvement at both the short- and long-term after BS and the precise mechanisms by which it can take place.
- Changes in the neuroendocrine system have also been suggested to be involved in the beneficial effects of BS. As summarized above, the principal hypotheses about beneficial neuroendocrine effects of BS include the modification of gastrointestinal hormone release with well-recognized effects on food intake and energy equilibrium. Most of these hormones not only communicate with the brain in an endocrine manner, but they also act in a paracrine fashion by interacting with specific receptors located on vagal afferent nervous fibers which innervate the gastrointestinal tract and are in close proximity to gastrointestinal endocrine cells (85, 86). In addition, vagal afferent endings also respond to mechanical stimuli such as distension (86, 87). The *nucleus tractus solitarius* (NTS; vagal afferent nucleus) senses afferent inputs to regulate energy balance, including hunger and satiety feelings by the activation of brain regions involved in food intake regulation, as well as by vagal efferent inputs that regulates splanchnic organs such as liver and endocrine pancreas (87–89). These pathways seem to be altered in obesity with vagal afferent over-activation likely due to a decreased sensitivity to stimuli of vagal afferents which requires higher stimuli to activate proper response to feeding which leads to hyperphagia (87). Accordingly, interventions aimed at both depletion or stimulation of vagal afferent fibers have been shown to improve weight loss and food intake control (90, 91). The fact that these opposite interventions as well as the existence of contradictory results regarding vagotomy suggests that the effect depend on the site where vagal afferents are intervened (92). Animal studies analyzing vagal afferent restructuring after BS support this hypothesis. Different vagal pathway reorganizations are induced after each bariatric procedure, but behavioral reduced food intake and increased satiety feelings are common after BS regardless the procedure (93). Both RYGB and SG involves the damage of gastric vagal afferent fibers but in different gastric regions (94). RYGB damages gastric branches close to their origin resulting in a decreased density of vagal afferents in the NTS, reduction of synapses and NTS microglia activation (95). It is noteworthy that other vagal afferent endings to the rest of the intestine and other splanchnic organs are preserved (96). By contrast, SG only damages terminal gastric vagal branches which oppositely results in increased density of vagal afferent in the NTS and the number of synapses, which has been suggested to be linked to higher sensitivity to nutrients and satiety feelings, and then to lower food intake (95). The fact that blocking of vagal afferent fibers in the band site abolishes AGB effects on food intake and satiety, highlights the role of vagal afferent pathways in mediating AGB effects likely by means of mechanoreception (97). Evidence from human studies dealing with the involvement of vagal system in BS effects are limited. Indirect evidence regarding how distension stimuli, and then mechanosensitivity by vagal afferent fibers, might be involved in the voluntary food intake reduction after RYGB and AGB has been reported (98, 99). Sundbom et al. used pancreatic polypeptide (PP) as marker for vagal efferent function after RYGB, finding a post-operatively decrease in ghrelin and PP associated with weight loss that authors attribute to vagal dysfunction (100). Accordingly, Tamboli et al. found an attenuation in PP response to ghrelin after RYGB (101). By contrast, Perathoner et al. did not find significant differences in satiety score, weight loss, motility or ghrelin and gastrin levels in RYGB patients with or without vagal nerve dissection (102). Although not having being directly related to vagal pathways, significant changes in neural response (specifically reduction of the activation of brain areas linked to the mesolimbic reward pathway) to food cues have been reported in morbidly obese patients undergone RYGB (103). Ten Kulve et al. suggested that the reduction in brain activation to food pictures and to the intake of palatable food in patients who underwent RYGB could be mediated by the post-operatively GLP1 increase, although precise mechanism linking GLP1 and brain activity response to food remains unknown (104). In view of these results, neural modifications that affect patient behavior regarding feeding habits could be induced by the different bariatric procedures. Nevertheless, the limited evidence in humans make difficult to confirm the relevance of these neural remodeling and whether are cause or consequence of BS effects.

BS also alters hormone levels other than incretins such as glucagon. Patients who underwent RYGB had decreased glucagon levels (105). This decrease was similar to the reduction due to general weight loss. However, other studies did not find a significant decrease in glucagon levels after other surgical procedures (106).

Obestatin levels have also been described to be implicated not only in food intake and gastric emptying but also to have beneficial effects on pancreatic β cells by reducing apoptosis and promoting proliferation, increasing insulin secretion and decreasing insulin resistance, which have a positive effect on systemic glucose homeostasis. It has been suggested that obestatin may play a role in T2DM remission (47).

Increased adiponectin levels after BS have also been described. Adiponectin is an anti-inflammatory hormone that improves insulin sensitivity, and its rise after BS has been correlated with higher T2DM remission rates in patients who underwent BPD (48, 49).

Despite the general overview that BS improves carbohydrate metabolism, there are also studies that have reported T2DM recurrence 5 years after surgery in 50–95% of patients who had achieved T2DM remission. This recurrence was associated with weight regain and a longer history of T2DM previous to the surgery (107, 108). Thus, more studies are necessary in order to provide clinical recommendations on the use of oral antidiabetic drugs in patients who have undergone BS with anatomical remodeling and the consequent physiological changes. Another relevant aspect that should be considered is neuroglycopenic hypoglycemia in BS patients, which could be related to the foregut hypothesis and low production of “anti-incretin” signals needed to counteract the dominant incretin action after surgery together with the hyperinsulinemia triggered by the increased incretin response in the distal intestine (55).

## Non-metabolic BS-Induced Endocrine Alterations

Aside from the well-known metabolic effects of BS, these surgical procedures also induce changes in other systems with further endocrine consequences (Table 1).

**Table 1:** Endocrine consequences of bariatric surgery on somatotropic, corticotropic, and gonadal axes.

		**Study**	**Surgery**	**Design**	**Follow-up**	**Sample size**	**Population**	**Primary outcome**	**Results**
**Somatotropic axis** *General trend*: Somatotropic axis restoration		Mittempergher et al. (109)	BPD LAGB	Observational, prospective study	1 year	88 (34/54)	Obese patient (male/female)	GH IGF-1	Higher effects of mainly malabsorptive techniques than restrictive techniques.
		Camastra et al. (110)	RYGB	Observational, prospective study	6 months	23 (16/7)	Severely obese and non-obese controls.	GH	Significant increase in GH secretion.
		De marinis et al. (111)	BPD	Observational prospective study	16 and 24 months	30 (15/15)	Obese females and non-obese females.	IGF-1 GH peak after GHRH	Slower IGF-1 secretion in response to BS possibly attributed to underlying catabolic status, as GH response to GHRH severely increased.
		Mancini et al. (112)	RYGB	Observational prospective study	6 months	10	Non-diabetic premenopausal severely obese women	GH	Partial recovery of somatotropic axis.
		Britt Edén Engström et al. (113)	RYGB	Observational prospective study	6 and 12 months	63 (54/9)	Obese patients (female/male)	GH IGF-1	Increase in GH and IGF-1 levels.
		Savastano et al. (114)	LAGB	Observational prospective study	6 months	254 (104/36)	Moderately and severely obese patients (female/male)	GH IGF-1 GH peak after GHRH plus arginine (ARG) test	Higher weight loss and improvement of body composition profile in subjects who recovered GH response to stimulus and with normal IGF-1 levels after surgery.
		Di somma et al. (115)	LAGB	Observational prospective study	6 months	72	Severely obese females	GH peak after GHRH plus arginine test. IGF-1 IGFBP-3	Postoperative IGF-1 levels were the strongest determinant of body composition profile. So, recovered GH axis is related with higher success of surgery.
**Corticotropic axis** *General trend:* Short-term: cortisol increase immediately after BS (acute stress) Long-term: corticotropic axis activation (although there are controversial results).		Manco et al. (116)	BPD	Observational prospective study	2 years	10	Fertile non-diabetic obese women	CBG, Plasma cortisol suppression with dexamethasone suppression test	A significant decrease in circulating CBG levels and an increase in the free cortisol fraction in obese women. No difference was found in cortisol suppression after BS.
		Morrow et al. (117)	RYGB	Observational prospective study	2 and 5 months	24 (10/14)	Obese patients with presence or absence of night-eating syndrome undergoing BS	Fasting plasma cortisol	Decrease in fasting plasma cortisol in obese patients without night-eating syndrome after BS.
		Larsen et al. (118)	LAGB	Cross-sectional study		34 (16/18)	Obese women with and without binge syndrome	Salivary cortisol	Lower salivary cortisol levels during the day in obese women with binge syndrome than without binge disorder.
		Guldstrand et al. (119)	LAGB	Observational prospective study	~12 months (“After a stable body weight after BS”)	8 (7/1)	Obese and non-diabetic patients (female/male)	Plasma cortisol	Reduction in cortisol levels in response to hypoglycemic clamp technique after BS-induced weight loss in comparison to the presurgical state characterized by exaggerated HPA axis activation.
		Ruíz-Tovar et al. (120)	SG	Observational prospective study	6 and 12 months	40	Morbidly obese patients	Serum cortisol, CRP	Cortisol levels decreased from 6 months after BS. CRP levels decreased significantly 12 months after BS.
		Valentine et al. (121)	BPD RYGB LAGB SG	Observational prospective study	6 and 12 months	24	Obese female participants	Salivary cortisol	A significant rise in morning salivary cortisol levels after BS, but no differences in nighttime salivary cortisol levels and the salivary cortisol awakening response.
		Hulme et al. (122)	RYGB LAGB SG	Observational prospective study	3 and 6 months	17 (14/3)	Obese patients (female/male)	Saliva Cortisol	No effect of BS on cortisol secretion daily patterns but morning cortisol showed a slightly non-significant increase.
**Gonadal Axis** General trends	*In women PCOS:* Improvement in gonadal function, menstrual irregularities and fertility. Androgen and hirsutism reduction.	Sarwer et al. (123)	RYGB LAGB	Observational prospective study	1 and 2 years	106	PCOS obese women	Total testosterone, estradiol, FSH, LH and SHGB levels	Significant improvements in general sexual quality, functioning and hormonal levels after BS.
		Jamal et al. (124)	RYGB	Observational prospective study	46.7 months	20	Obese female with ≥ 2 of 3 diagnostic criteria for PCOS	Hormonal levels (Testosterone, SHBG, LH, FSH, estradiol levels), menstrual cycles, hirsutism	An improvement in gonadal dysfunction in 82% of patients with a recovery in menstrual irregularities, 89% hirsutism resolution, and 50% achieve conception.
		Eid et al. (125)	RYGB	Observational prospective study	27.5 ± 16 months	24	PCOS obese women	Menstrual cycles, hirsutism, hormonal levels	Improvements in PCOS-associated symptoms including menstrual alteration resolution, and hirsutism. Successful conception was achieved by 5 patients.
		George and Azeez. (126)	SG	Retrospective analysis		132	PCOS Obese women	Clinical dates: menstrual cycles, hirsutism, hormonal levels and radiologic ovary pattern	Resolution of menstrual irregularities pattern in the majority of the cases, of hirsutism in 80% and of the radiologic pattern in PCOS in 81%.
		Skubleny et al. (127)	BPD RYGB LAGB SG	Meta-analysis	1-year	2130	PCOS Obese women	Hormonal levels and clinical sequelae of PCOS: menstrual cycles, hirsutism, and infertility.	PCOS significantly decrease from 45.6% pre-operatively to 6.8% 1 year post-operatively.
		Shekelle et al. (128)	BPD RYGB LAGB SG	Meta-analysis of cohort studies, case series and individual case reports.		57 articles analysis	Obese and reproductive age women	Fertility, contraception, pregnancy, weight management, and nutritional deficiencies.	Menstrual regularity was recovered in 71%; with an association between weight loss and ovulation recovery. Data suggest improvement fertility after BS with minimal nutritional deficiencies for mother and child and without higher complications in post-surgery pregnancies.
	*In MOSH men:* MOSH is supposed to be a reversible situation in the majority of cases with normalization of serum testosterone levels, fertility improvement in male severe obesity, but there are discrepancies about spermatogenesis.	Reis et al. (129)	RYGB	Prospective randomized controlled trial	24 months	20 (10/10)	Obese men in 2 groups with life style modification and RYGB	IIEF test, serum estradiol, PRL, LH, FSH, free and total testosterone	Improvements in sexual functioning and hormonal levels (total testosterone, FSH and PRL).
		Mora et al. (130)	RYGB SG	Prospective observational case series study	1 year	39	Obese men	IIEF score, Testosterone, SHBG, estradiol, gonadotropins, inhibin B, PRL.	Improvement in sexual aspects (IIEF score and significant increment in testosterone level).
		Sarwer et al. (131)	RYGB	Prospective cohort study	4 years	32	Obese men	SHBG, IIEF, Testosterone	Increase total testosterone and SHBG levels 4 years post-operatively, but improvements in sexual dysfunction were not significant during the follow-up.
		Facchiano et al. (132)	BPD RYGB LAGB	Prospective study	6 months	20	Obese men	LH, FSH, Total and Free testosterone, SHBG, estradiol,	Increase in total testosterone, SHBG, LH and FSH levels with a relevant drop in estradiol levels.
		Luconi et al. (133)	BPD RYGB LAGB	Longitudinal study	6 and 12 months	24	Morbidly obese male	Free-testosterone, SHBG, LH, FSH	Increase in total and free testosterone levels as well as SHBG and gonadotropins (simultaneous increases in LH and FSH).
		Aarts et al. (134)	RYGB LAGB	Observational study	1-year	24 (13/11)	MOSH and eugonadal Obese men	Free-testosterone	Increase in free-testosterone in both MOSH and eugonadal groups.
		Samavat et al. (135)	BPD RYGB LAGB SG	Cohort study		55 (29/26)	Morbidly obese men (with MOSH and 26 without)	Total testosterone; Free testosterone; Gonadotropins. SHBG and estradiol levels.	Increase in androgen levels (total and free-testosterone) only in patients with hypogonadism. Decreased estradiol levels only in eugonadal patients. MOSH reversal that occurred early after surgery and was nearly complete.

*BPD, Biliopancreatic diversion; SG, Sleeve gastrectomy; RYGB, Roux-en-Y gastric bypass; LAGB, Laparoscopic adjustable gastric band; IGF-1, Insulin-growth factor-1; GH, Growth hormone; GHRH, Growth hormone-releasing hormone; CBG, Corticosteroid-binding globulin; CRP, C-reactive protein; HPA axis, Hypothalamic-pituitary-adrenal axis; BS, bariatric surgery; PCOS, Polycystic ovary syndrome; MOSH, Obesity-associated secondary hypogonadism; IIEF, International Index of Erectile Function; PRL, Prolactin; LH, luteinizing hormone; FSH, Follicle-stimulating hormone; SHBG, Sex hormone binding globulin*.

### Somatotropic Axis: Growth Hormone (GH)/Insulin-Growth Factor-1 (IGF-1) After BS

GH is mainly regulated by two hypothalamic peptide hormones: GH-releasing hormone (GHRH) and somatostatin. However, other brain signaling pathways such as those related to sleep regulation are also involved in the regulation of GH secretion (136). Obesity is associated with an acquired functional reduction in GH secretion and in GH response to stimulus (e.g., insulin-induced hypoglycemia, arginine, arginine-GHRH, sleep, or exercise), which is reversible after significant weight loss (137, 138). Likewise, adiposity acts as a negative determinant for GH secretion frequency and width, and has been associated with an increase in GH removal, which implies a lower GH half-life (109). A combination of multiple somatotropic axis alterations might be responsible for the different grades of GH and IGF-1 deficiency in obese subjects. However, the physiological role of GH in obesity and the mechanisms that lead to the perturbations in its levels are not completely clear. Among the underlying neuroendocrine alterations of low plasma GH levels in obesity, hormonal GHRH, somatostatin, or ghrelin dysregulation have been proposed (136, 139). It should be also noted that gender is a relevant regulatory factor of GH secretion in healthy subjects (113).

Reports regarding serum IGF-1 levels in obesity have shown contradictory results with both normal (109, 140, 141) and decreased IGF-1 levels in obese subjects having been described (142). Recent studies have suggested that inflammatory mediators could play a relevant role in the decrease in IGF-1 bioactivity (143), which could be related to obesity which is characterized by a chronic low-grade inflammation. Obesity, specifically abdominal obesity, appears to be associated with a decrease in free IGF-1 levels. This finding is highly interesting since visceral fat mass plays a key role in the development of obesity-related diseases, and low IGF-1 levels have also been related to T2DM and CVD (144, 145).

Somatotropic axis alterations in obese subjects, similar to those in patients who have a GH deficit, are associated with a higher prevalence of cardiovascular risk factors and alterations in body composition (muscle mass and bone density reduction and rise in fat mass) (146–148). Concordantly, Di Somma et al. found that obese patients with severe somatotropic axis alterations showed an exacerbated cardio-metabolic risk with more serious sequelae compared to obese subjects without GH deficiency (114).

Several studies have reported a restoration of somatotropic axis alterations in severely obese patients after BS. A significantly increased secretion of GH after BPD has been described (110), as well as partial recovery of the somatotropic axis after RYGB (112). Mittempergher et al. determined that the GH increase after BS was higher after mainly malabsorptive procedures (2.7-fold increase) than after mainly restrictive procedures (1.4-fold increase) with parallel improvements in cardiovascular profile and BMI reduction. Therefore, in view of these results, malabsorptive procedures might be better recommended for patients with somatotropic deficiencies (109). However, further research is necessary in order to confirm these recommendations.

Britt Edén Engström et al. determined that GH secretion and IGF-1 levels, previously compromised in severe obese subjects, were augmented at 6 months in women and at 12 months in both women and men after RYGB. A concomitant BMI reduction was reported in men and women at 6 months, which was further improved at 12 months after surgery (113). Savastano et al. and Di Somma et al. reported significant correlations between body composition and GH response to stimulus (GHRH + arginine) before and after surgery in subjects who underwent LAGB, and also found higher success with surgical treatment (weight loss and improved body composition profile) in those subjects without somatotropic axis alterations as well as in patients who recovered GH response to stimulus after surgery (114, 115). BS-derived benefits can be influenced by presurgery GH response to stimuli and by previous IGF-1 levels adjusted for sex and age. Thus, these factors could be interesting markers of surgery success in weight loss and body composition improvement. Likewise, a somatotropic axis study may constitute a useful tool for obese patient follow-up after BS (115).

Although some discrepancies have been found in the literature (109, 149), IGF-1 secretion displays a slower response to BS, similar to that of non-surgical weight loss, and this can be related to an underlying catabolic status induced by BS (malabsorptive effects, pronounced restriction in caloric intake, etc.) (111, 113). Few studies have analyzed tissue response to GH in obesity. Some have described a more pronounced IGF-1 response in obese individuals compared to lean subjects (150, 151). Nevertheless, these findings were not confirmed by others (152). Britt Edén Engström et al. observed that most obese subjects showed high IGF-1 levels with regard to their GH status. Altogether, these findings may indicate that there is a specific tissue adaptation in obesity with higher GH sensitivity, especially to its anabolic effects (113). In view of these results, the acquired dysfunction in GH secretion secondary to obesity appears to be reversed after BS. The somatotropic axis status could constitute a useful tool for initial and post-surgery follow-up of BS patients as a marker of surgery success in terms of weight loss and body composition.

### Hypothalamic-Pituitary-Adrenal (HPA) or Corticotropic Axis: Corticotropin-Releasing Hormone (CRH)/Corticotropin (ACTH)/Cortisol After BS

The HPA axis is a relevant pathway in the response to physiological stress which regulates cortisol secretion. Cortisol has a number of effects on the organism, including energy reserve mobilization to promote survival and to comply with metabolic requirements during stress. Cortisol is secreted by the suprarenal cortex after HPA axis activation in response to physiological or psychological stress and high cortisol levels are associated with intense emotional responses to stressful stimuli (153). However, cortisol metabolism is not only centrally regulated, but type 1 and 2 11-β- hydroxysteroid dehydrogenase enzymes also regulate cortisol in tissues (154, 155). Food intake also increases cortisol levels and an individual may feel a decrease in anxiety with eating (156). Thus, both high cortisol levels and distressed emotions, appear to be modulated by calorie-rich food intake. Once this neuroendocrine-behavioral response to chronic stress is initiated, it could become a habit that favors weight gain. The non-pathological HPA axis is characterized by a distinctive circadian profile that is disrupted when the HPA axis is deregulated (157, 158). The declining curve of daily cortisol secretion is usually less pronounced in chronic health disorders such as obesity (159, 160). During chronic stress, HPA axis adaptation results in a higher and more prolonged cumulative exposure to glucocorticoids, which are associated with negative effects including metabolic disorders and cardiovascular mortality (161–166). Emerging data suggest that metabolic syndrome patients could have a hyperactive HPA axis which would lead to “functional hypercortisolism.” However, there are some controversial and heterogeneous findings in the studies analyzing this relationship (154, 155, 167). Moreover, there are disorders associated with HPA axis dysregulation that are more highly prevalent among obese subjects compared to the general population such as night-eating syndrome, binge eating disorder, sleep, and hormonal disorders as well as psychological symptoms (168–171). It is worth of mentioning that treatment for weight loss in obese subjects with these disorders is less successful than in obese individuals without these types of disorders (168, 172).

Very few studies have examined HPA axis regulation before and after BS in humans. The most commonly held idea is that weight loss tends to normalize cortisol levels and possible alterations in the HPA axis (173). Nevertheless, some studies suggest that long-term weight loss could be lessened when methods based on caloric restriction are employed, since caloric restriction could activate the HPA axis and increase circulating cortisol levels and these are associated with higher food intake and body weight (174–176). An increase in HPA axis tone during weight loss through food restriction can weaken behavioral modifications, which would make patient compliance to the prescribed diet and sustained weight loss in the long term difficult (177–179). Current evidence suggest that BS can affect HPA axis regulation, but controversial results have been found regarding the direction of this regulation (116, 119–121). Cortisol increases immediately after BS due to the acute stress caused by the surgery itself (180). However, the long-term effects of BS on cortisol have not yet been elucidated. Moreover, the few studies dealing with HPA axis after BS used different methodology for assessing HPA axis, which makes difficult to have clear conclusions regarding the effect of BS on HPA axis.

Morrow et al. studied HPA axis modulation in 24 severely obese participants (BMI = 40–70 Kg/m^2^) without associated comorbidities who underwent RYGB. Participants were classified according to the presence or absence of night-eating syndrome. A decrease in fasting plasma cortisol levels 5 months after surgery was reported in patients without night-eating syndrome, but an increase was seen in the subgroup of patients who had night-eating syndrome. However, the two groups did not differ in weight loss or waist circumference (117). By contrast, Hulme et al. found that BS did not affect daily cortisol secretion patterns during the first 6 months after surgery, although morning cortisol showed a slightly non-significant post-operative increase (122).

Valentine et al. also found a significant rise in morning saliva cortisol levels 6 and 12 months after BS (54.2% after SG; 16.7% after LAGB; 12.5% after RYGB, and 8.3% after duodenal switch) in 24 obese women, but no differences were found in nighttime saliva cortisol levels or in salivary cortisol awakening response (121). High morning cortisol could be associated with an improvement in physiological health after BS that is expressed by the narrowing of the decreasing curve of diurnal cortisol secretion in contrast to plainer curve characteristics in chronic health disorders.

Larsen et al. analyzed salivary cortisol levels in obese women after LAGB and found that neuroendocrine regulation after BS differs in these patients depending on the presence or absence of binge eating disorder. Obese women with binge eating disorder had significantly lower salivary cortisol levels during the day than patients without binge disorder. It is of note that a normal weight control group was not included in this study (118).

Manco et al. described a significant decrease in circulating corticosteroid-binding globulin (CBG) levels and a concomitant increase in the metabolically free cortisol fraction in obese women 2 years after BPD (116). The increase in free cortisol fraction may contribute to adaptive enterocyte hyperplasia in the long term, favoring better functional morphology as cortisol is a regulator of crypt enterocyte proliferation (181). Likewise, hypophyseal response, and/or suprarenal sensitivity, assessed by intravenous dexamethasone infusion, differed between the patients who underwent BPD and the normal weight control group. Nevertheless, there were no differences in cortisol suppression induced by dexamethasone before and after BS (116).

Guldstrand et al. studied HPA axis changes after LAGB in 8 non-diabetic severely obese patients. For this purpose, cortisol levels were analyzed in response to hypoglycemic clamp technique and a reduction in counter-regulatory hormone (cortisol among them) response was observed during the sustained hypoglycemic state after BS-induced weight loss in comparison to the pre-surgical state characterized by exaggerated HPA axis activation (119).

Ruiz-Tovar et al. reported sustained high serum cortisol levels up to 6 months after BS in 40 morbidly obese patients who underwent laparoscopic SG. However, cortisol levels decreased and were directly associated with the CVD risk predictor triglyceride/HDL ratio from 6 months after BS (120).

In view of these studies, obesity-induced corticotropic axis activation is reduced after BS and a normalization in HPA circadian rhythms might be also occurring. This effect has not been described for caloric restriction, which could add value to the use of BS for obesity treatment. However, although current evidence suggest that BS can affect HPA axis regulation with improvement in axis physiological health, controversial results have been found regarding the direction of this regulation. Therefore, more studies on this issue are needed to homogenize criteria concerning which variables should be used to monitor corticotropic axis status.

### Modifications in Gonadal Axis or Hypothalamic/Pituitary/Gonadal Axis: Gonadotropin-Releasing Hormone (GnRH)/Follicle-Stimulating Hormone (FSH), Luteinizing Hormone (LH)/Testosterone, and Estradiol After BS

The incidence of sexual dysfunction, reproductive disorders, and infertility increase with age. The relationship between sexual dysfunction and other medical conditions such as obesity (182), diabetes (183, 184), high blood pressure (185, 186), metabolic syndrome, CVD or smoking (187) is widely accepted.

It is also well-known that adipose tissue plays a relevant role in the metabolism of hormones, including sex hormones, secreted by different glands (188). Obesity-associated gonadal dysfunction is one of the most prevalent comorbidities in obese subjects who seek to lose weight (29% for women and 45% for men) (189). These disorders are particularly common in severely obese subjects who have undergone BS (190). Similar to other obesity comorbidities, the higher the BMI, the higher the risk of gonadal dysfunction (191–193). A number of recent studies have dealt with this issue. Sarwer et al. studied gonadal dysfunction prevalence in 250 obese patients (141 who were interested in BS and 109 in lifestyle changes) finding differences according to the preferred treatment for weight loss achievement. Among individuals interested in BS, 51.4% of women (according to the Female Sexual Function Index) and 36.4% of men (according to the International Index of Erectile Function) had sexual dysfunction. By contrast, 40.9% of women and 20% of men who sought lifestyle modifications had sexual dysfunction. These differences were mainly related to BMI discrepancies. Sexual dysfunction was associated with quality life and other psychosocial functions, particularly in women (192). Interestingly, the significant improvement found in sexual functioning occurred mainly in women (130, 187, 194).

It has been widely described that BS leads to an improvement in sex hormone and sexual hormone binding globulin (SHBG) levels in morbidly obese patients (132, 133, 193, 195), even higher than the effect attributable to weight loss alone (133). Thus, this supports the hypothesis that factors derived from excess adipose tissue may modulate the gonadal axis (196). An increase in SHBG and a modification in serum estradiol levels are detected after BS in men and women. This could be due to the effect on estrogen production from testosterone and steroid precursors mediated by aromatase (an enzyme whose higher activity has been associated with gonadal dysfunction pathology in obesity) in reduced adipose tissue after BS (197).

The main evidence found in this context are due to: (1) excess androgen in obese women, mainly associated with polycystic ovary syndrome (PCOS) (36%) and with idiopathic hyperandrogenism (198), and (2) androgen deficiency in obese men that is termed male obesity-associated secondary hypogonadism (MOSH). A recent meta-analysis highlights that PCOS and MOSH are among the most common comorbidities in severely obese patients who have undergone BS (36 and 64%, respectively) that are usually resolved after BS (96% of women with PCOS and 87% of men with MOSH), as highlighted by a recent meta-analysis (191).

This improvement in gonadal profile occurs in parallel with insulin resistance improvement and metabolic disorder resolution (191).

Sex-specific changes induced by BS have been reported as described below.

#### Modifications in the Gonadal Axis in Obese Women After BS

Excess adipose tissue in obese women may contribute to excess androgens by stimulating both ovary and hormonal secretion by the suprarenal glands as secondary effects of insulin resistance and compensatory hyperinsulinism. Specifically, abdominal adiposity may feed a vicious circle in which excess androgens favors body fat deposition and this visceral fat promotes ovary and adrenal-derived excess androgens in PCOS. However, for PCOS to develop in response to obesity and visceral fat accumulation, women must have a primary defect in steroidogenesis that promotes excess androgen secretion, predisposing to androgen excess disorders (199). In contrast, women without this primary defect in androgenic secretion do not develop excess androgens or PCOS, even in the case of extreme obesity or insulin resistance (198). Renowned authors in the field consider that obesity is one of the secondary phenotypes of PCOS, and in these cases, gonadal dysfunction improves and even resolves with weight loss (200). A recent meta-analysis has demonstrated that PCOS significantly decreased from 45.6% pre-operatively to 6.8% 1 year after surgery (127). A decrease in serum androgen levels (total testosterone) associated with a reduction in androsterone and sulfate dehydroepiandrosterone (DHEA) as well as with resolution of hirsutism and menstrual irregularities (53 and 96% of cases, respectively) has been reported. Improvements in estradiol, FSH and SHBG have also been described (191, 193, 201–203).

Studies dealing with fertility recovery in women with PCOS that have assessed fertility before and after BS are scarce. The existing studies suggest that female fertility improves after bariatric procedures and intensive weight loss. However, most of these are observational studies with high variability in age, in the bariatric procedure employed and without a control group. This makes reach a consensus about the role of BS in fertility management difficult. Accordingly, smarter studies with a precise design comparing the different bariatric procedures are required (204). Among the studies dealing with fertility, Eid et al. demonstrated that RYGB-induced weight loss was associated with improvements in PCOS-associated symptoms including menstrual alteration resolution in all the participants and hirsutism in half of the participants. In addition, successful conception was achieved by five patients who previous to surgery failed to conceive (125).

George and Azeez carried out a study that included 132 women with PCOS who underwent SG in whom resolution was achieved in the majority of the cases of menstrual irregularities, hirsutism (80%) and radiologic irregularities (81%) (126).

Jamal et al. also observed improved gonadal dysfunction in 82% of RYGB PCOS patients, with decreased menstrual irregularities and resolution of hirsutism in 29% of the patients, and 50% of patients who were unable to conceive prior to surgery were successful after surgery (124).

Balen et al. based on low-quality evidence, recommended BS use in obese PCOS women with a BMI>35 kg/m^2^ and comorbidities such as gonadal dysfunction or BMI>40 kg/m^2^ who had infertility problems despite programmed intensive and structured treatment (based on dietary-hygienic habit changes) with no response for at least 6 months. They also recommended to avoiding pregnancy in the rapid post-operative weight loss period for at least 6–12 months after BS and at later times, recommended that these women should be attended in a specialized multidisciplinary unit due to the risk of BS-associated nutritional deficiencies (205).

Although PCOS is the most frequent reproductive disorder in obese women (191), idiopathic hypogonadotropic hypogonadism has also been associated with higher obesity prevalence in the female population (206). Several studies have reported improvements in the gonadotropic axis as well as sexual functioning after BS in obese women that are not necessarily related to PCOS. Sarwer et al. reported that after BS, 106 women (80% RYGB and 20% LAGB patients) showed significant improvements in general sexual quality and functioning as well as sexual desire and satisfaction 1 year after surgery. These improvements were maintained up to 2 years after BS. These changes took place in parallel with psychosocial improvements, significant weight reduction of 32.7% and significant improvement in hormonal profile: decreased total testosterone, estradiol and DHEAS and increased FSH, LH and SHGB levels (123).

Bond et al. described that in 68% of obese women with sexual dysfunction (defined by the Female Sexual Function Index score), this condition resolved after BS. This improvement in sexual function was not dependent on type of surgery or amount of weight loss and appears to be an additional benefit for women undergoing BS (194).

As detailed above, weight loss after lifestyle modification, and particularly after BS, is associated with significant improvements in sexual functioning and resolution of both menstrual and ovulatory disorders that occur in nearly all patients. Thus, weight loss could also contribute to fertility restoration (191, 194, 201). However, Shekelle et al. pointed out in their review that most of the reports are observational studies and data should be interpreted cautiously (128). Moreover, except for PCOS, the definition of pre-operative reproductive disorders in obese female patients having undergone BS is not clearly defined. It is of interest to understanding whether results of these reports are referring to PCOS or to different and independent reproductive anomalies to confirm whether BS is also effective for the resolution of additional reproductive disorders.

#### Modifications in Gonadal Axis in Obese Men After BS

In obese men, excess adipose tissue appears to contribute to androgenic deficiency by means of pituitary gonadotropin inhibition. This suggests central production deterioration together with reduced gonadal sensitivity to LH and increased peripheral androgen degradation by their conversion to estrogens (207, 208). MOSH is characterized by reduced serum testosterone together with high relative estradiol levels and inappropriately low or normal levels of LH and FSH (209). Although serum LH levels and the pulse frequency are similar in obese and lean subjects, LH pulse width is noticeably reduced in obesity (210). Among mechanisms involved in MOSH pathogenesis, gonadotropin inhibition by excess peripheral estrogen production in adipose tissue has been reported. However, insulin resistance and the secretion of inflammatory mediators and adipokines may play a relevant role in MOSH physiopathology with an inhibitory action at the hypothalamic-pituitary level (196, 211, 212). Recent data suggest that not only hyperinsulinemia but also excess liver fat are among the main inhibitory influences on SHBG secretion in obese subjects, in a process in which pro-inflammatory cytokines derived from ectopic adipose tissue are likely implicated (213). Likewise, hypophyseal dysfunction induced by obstructive sleep apnea syndrome may also participate in MOSH development (214).

Thus, abdominal adiposity could favor a vicious cycle in MOSH development by inhibiting hypophyseal gonadotropin secretion (215–217) and simultaneously, the resulting androgen deficiency promotes body fat accumulation in parallel to a decrease in muscle and lean mass (218).

Substantial fat mass loss after intensive weight loss will likely tend to reduce aromatase activity and regulate estradiol/testosterone equilibrium, resulting in decreased estradiol production, and will favor LH increase which contributes to stimulating testosterone production and substrate availability for estradiol production. It is thought that the differences between obese subjects with normal gonadal function and those with hypogonadism might be determined by aromatase expression levels: MOSH would be induced when aromatase gene expression is elevated. By contrast, testosterone levels would remain within the normal range when aromatase expression levels are low (134). Similar to PCOS in obese women, MOSH is not a generalized condition in male obesity and underlying mechanisms are largely unknown.

An increase in serum androgen levels (for both total testosterone and the free testosterone index), which was even higher than the increase in SHBG levels in men has been reported (134, 191). Normalization of serum testosterone levels after BS is associated with improved fertility in severely obese men. Nevertheless, more specific fertility studies focused on spermatogenesis and androgen levels are needed given the discrepancies between the studies published to date (191). Some studies suggest a deterioration in seminal parameters (219, 220), while others have observed no effect (202, 221) or even beneficial effects on sperm quality (222). It has also been postulated that fat mass reduction associated with weight loss might reduce abdominal, thigh and scrotal fat depots. This leads to a decrease in testicular temperature and, consequently, facilitate spermiogenesis (207).

Chronic testosterone deficiency could reduce post-operative fat loss and enhance the catabolic effects of BS on muscle and bone. This would attenuate the positive effects of BS on insulin sensitivity. A number of recent studies dealing with the changes and benefits in gonadal dysfunction due to BS in obese men, are in agreement with BS-induced improvements in testosterone, SHBG and LH levels (129, 223, 224). Although diet and lifestyle changes and BS are associated with a significant increase in hormone levels, MOSH normalization (increased testosterone and gonadotropin levels together with decreased estradiol levels) was more effective with BS than with low-caloric diets. By contrast, results regarding FSH levels are more controversial (129, 193, 223).

Reis et al. found significant improvements in sexual functioning and levels of hormones such as total testosterone, FSH and prolactin in 10 RYGB patients (129).

Mora et al. observed a pronounced weight loss (77.18% of excess weight), metabolic profile improvement together with improvements in sexual aspects such as an improved score on the International Index of Erectile Function and a significant increase in testosterone levels 1 year after surgery (RYGB and SG in 46.2 and 53.4%, respectively) in a prospective study carried out in 39 obese men (130).

In agreement, Sarwer et al. reported a concomitant decrease in patient weight by nearly one third and increase in total testosterone and SHBG levels 4 years after surgery in their cohort of 32 men who underwent RYGB and who showed deficient sexual functioning prior to surgery. Despite this finding and although improvements in sexual dysfunction were seen in the short term after surgery, these changes were not significant during the follow-up (131).

Facchiano et al. reported an increase in total testosterone, SHBG, LH, and FSH levels with a relevant drop in the initially elevated estradiol levels at 6 months after BS (10 with RYGB, 8 with LAGB and 2 with BPD) in 20 obese men (132).

The findings of Luconi et al. confirmed the already known relationship between excess adiposity and hypogonadism in morbidly obese men. In the same line as previous studies, patients showed increased total and free testosterone levels as well as gonadotropins (simultaneous increases in LH and FSH). The increased testosterone and SHBG levels induced by BS were higher than those expected for the weight loss alone. Likewise, the increase in total testosterone may be related to an SHBG rise and peripheral aromatization reduction that contributes to the observed fall in estradiol levels. Moreover, the direct effect of weight loss on Leydig cell function should not be discarded in the improvement of gonadal profile (133).

Aarts et al. included 24 severely obese men (BMI = 35–59 Kg/m^2^) in their study, 13 of them with MOSH and 11 eugonadal morbidly obese patients. One year after BS, an increase in free testosterone was observed in both MOSH and eugonadal groups. These levels were inversely related to weight loss. MOSH was a reversible situation in the majority of cases and this pre-operative condition was not adversely associated with BS efficiency in terms of weight, lipid and carbohydrate metabolism or with catabolic effects of surgery on muscle or bone mineral density (134).

Nevertheless, Samavat et al. in their study with morbidly obese BS patients (29 with hypogonadism and 26 eugonadal patients) observed that presurgery testosterone levels appear to moderate the BS effect on sex hormone recovery as an increase in androgen levels (total and free testosterone) only occurred in patients with hypogonadism. Preoperative estrogen levels were significantly lower in hypogonadal patients than in eugonadal patients. Moreover, estrogen levels only decreased after BS in eugonadal patients. Estradiol reduction in eugonadal patients was not related to testosterone level increment. As in the previous studies referenced above, Samavat et al. found a hypogonadism reversal that occurred early after surgery and was nearly complete. Taking into consideration these findings, the role of estradiol in obesity-related hypogonadism development might be limited (135).

All the revised studies, except for the study by Leenen et al. (225), reported a significant increase in total testosterone levels with weight loss and visceral fat reduction.

Few long-term studies have focused on the possible recurrence of gonadal dysfunction after weight regain. Rosenblatt et al. reported that patients who underwent RYGB from 6 to 16 years ago had high SHBG, total and free testosterone levels compared to non-surgical obese controls and these levels were comparable to those found in lean subjects. These findings agree with androgenic normalization in the long term. Nevertheless, the erectile function score was lower than in lean controls, which suggests incomplete functional restoration that may be related to weight gain and recurrent obesity comorbidities (226).

Recent research suggests reciprocal control between bone and testicles by means of osteocalcin (a peptide secreted by osteoblasts). It has been recently proposed that this peptide may be implicated in male fertility through direct stimulation of testosterone production by Leydig cells (227–231). Osteocalcin is decreased in obesity but it can also be produced by adipose tissue apart from bone production (232). Therefore, the inverse relationship between BMI and osteocalcin levels could indicate that only healthy adipose tissue may be involved in osteocalcin production (233). Weight loss induced by caloric restriction or BS is associated with the recovery of osteocalcin production. Samavat et al. analyzed 103 obese men, 76 patients who underwent RYGB and 27 controls who were waiting for surgery. The RYGB group showed an increase in osteocalcin, total testosterone and SHBG levels concomitant to a slight rise in gonadotropin levels, while estradiol levels fell likely due to a reduction in aromatase activity or expression. Osteocalcin levels were significantly associated with the restoration of testosterone levels in MOSH patients. Thus, osteocalcin may be an independent predictor for androgen restoration. It should be noted that no significant changes were seen in the non-surgical control group that had a slight weight loss. The osteocalcin levels increase might be explained by changes in leptin and adiponectin levels that regulate bone turnover in opposite ways. Likewise, insulin sensitivity improvement may enhance osteocalcin bone release (234). A recent report from Karsenty et al. supports the existence of a bone-testicle axis which is parallel to the pituitary-gonadal axis. In this way, osteocalcin and LH would act in parallel in both axes and osteocalcin would also be involved in human reproduction function (235).

In view of these results, BS is an effective method to treat gonadal dysfunction including recovery of fertility in comparison with lifestyle changes and diet-induced weight loss (200, 236, 237). Infertility or gonadal dysfunction could be used as criteria for indication for BS in obese patients with a BMI > 35 kg/m^2^. The considerable resolution rate of PCOS and MOSH, the most representative gonadal dysfunction disorders in obese individuals, after BS consistently supports the causal relationship of both obesity and adipose tissue dysfunction with gonadal dysfunction in predisposed subjects. However, more long-term studies focusing on the persistence of gonadal dysfunction resolution after BS are required.

### Modifications in Bone Metabolism After BS

It has been widely described that obese patients are at higher risk of vitamin D deficiency pre-operatively, although it is not clear whether this association is more closely related to obesity *per se* or to obesity-related diseases such as T2DM (238). Consequently, secondary hyperparathyroidism due to vitamin D deficiency has also been extensively reported in obesity (239). Paradoxically, obese patients are generally characterized by higher bone mineral density (BMD) and bone mineral content (BMC), and it has even been suggested that obesity could be protective against osteoporosis (240). Bone remodeling implies a continuous process of bone replacement (formation) and bone removal (resorption). When these two processes are unbalanced, net bone formation or bone loss, respectively, will occur (241). Bone remodeling not only promotes bone repair, but also helps to mechanical stress adaptation. Thus, mechanical bone loading determines bone strength, size, and mass. This has led to the suggestion that mechanical bone loading could be responsible for the protective role of obesity in bone health. Larger bone size (that can also be due to increased mechanical loading) and enhanced aromatase activity from adipose tissue have also been suggested factors favoring BMD in obesity (240–242).

The most common nutritional deficiencies after BS include folate, iron, vitamin B_12_, calcium, vitamin D and zinc. Therefore, post-surgical changes in calcium and vitamin D can lead to bone loss resulting in higher fracture risk (243). In fact, it has generally been suggested that BS negatively affects bone health by diminishing BMD, enhancing bone resorption and impairing even further vitamin D levels and hyperparathyroidism (244). Most of the evidence comes from RYGB studies with fewer studies on other types of BS, but also finding negative effects from mainly restrictive procedures (245). One of the hypotheses posited to explain BMD and BMC after BS is mechanical unloading. A lower mechanical load due to weight loss can lead to less bone formation followed by a decrease in BMD. Concordantly, RYGB is associated with bone loss as reflected by BMD reduction at several sites 6 months (246), 1 year (247–251), and 2 years after surgery (22, 243, 252). Several studies have been carried out with longer follow-up periods up to 6 years finding a persistent bone loss (253–256). BPD has also been related to a higher incidence of metabolic bone disease 1–5 years after surgery (257) and Tsiftsis et al. reported decreased lumbar spine BMD 1 year after surgery (258). This BMD decrease could be site dependent since Marceau et al. found no changes in BMD in the hip but did find a decrease in the lumbar spine (259). Prospective studies comparing BMD at different sites between gastric bypass and SG, found that both procedures led to decreased BMD in the lumbar spine and femur. This decrease tended to be less pronounced after SG in most of the studies (260–262), but comparable in others (254, 263). Pluskiewicz et al. also reported a reduction in BMD in the lumbar spine, femoral neck and hip 6 months after SG (264). Results from LAGB are even more scarce. Decreased BMD and BMC have been reported 1 and 2 years after LAGB (265, 266). This negative effect seems to be smaller than that observed after RYGB (23). The effects of LAGB on BMC were compared to diet-induced weight loss and similar results were found (267). By contrast, Mach et al. did not find significant changes in BMC 1 year after LAGB (268).

BMD and BMC are determined by means of dual-energy X-ray absorptiometry (DXA), and technical limitations should be considered when data are interpreted. DXA are technically limited when applied to obese patients, particularly in severely obese subjects. Modifications in fat mass distribution due to drastic weight loss could influence measurement accuracy (22, 241, 269). Therefore, the controversial results regarding the effect of the different bariatric procedures on BMD and BMC could be due to technical issues.

In addition, it is unclear whether the reported BMD and BMC decrease after BS, is clinically relevant regarding osteoporosis incidence and fracture risk. As recently reviewed by Ben-Porat et al. different studies have found controversial results (245). In a study with a large sample size (*n* = 2079) in which most of the patients underwent LAGB, the risk for fracture was not increased 2 years after surgery (270). Nevertheless, when most of the population underwent RYGB, an increased fracture risk was observed in a retrospective study with a median follow-up of 7 years (271). These results were replicated after gastric bypass for a median follow-up time of 3.1 years (272). Studies that compared BS patients to obese and control patients, found higher fracture risk 4 years after surgery in the BS group than in the two control groups, significantly so with BPD. After 12 years there was also higher fracture risk after mainly malabsorptive procedures (273). A recent meta-analysis that included six studies concluded that BS is associated with increased fracture risk compared to control populations. However, the length of follow-up and the bariatric procedures were heterogeneous (274). Lack of long-term prospective studies does not help to elucidate the real effect of BS on osteoporosis development and fracture risk (245, 269).

The serum 25(OH)D form of vitamin D, calcium and parathyroid hormone (PTH) have also been measured to verify putative effects of BS on bone metabolism. As calcium is preferentially and actively absorbed in the proximal gut, and pancreatic secretion and bile acids are required for vitamin D absorption, bariatric procedures that bypass the duodenum and proximal jejunum could promote malabsorption of these micronutrients. Decreased stomach acid after SG and bypass of the proximal bowel would also reduce calcium absorption (22, 243). A meta-analysis analyzing 10 gastric bypass studies found, in addition to BMD reduction, a significant decrease in serum calcium levels (275). BPD was also associated with an increased prevalence of hypocalcemia (257). Gehrer et al. however, found no significant effect of RYGB or SG on serum calcium levels (276). Serum calcium levels are tightly regulated. While the intestine is responsible for calcium absorption from the diet, calcium reabsorption occurs in the kidneys. When these two mechanisms are insufficient to maintain serum calcium levels, calcium is released from the bones, which act as a calcium reservoir. Thus, despite normal calcium levels, low BMD could be present (22, 243). A more accurate way to evaluate calcium alteration after BS could be the direct determination of calcium absorption. Schafer et al. and Riedt et al. did this and found that gastric bypass led to a decline in calcium absorption 6 months after surgery (277, 278).

The tight regulation of serum calcium levels relies on PTH and the active form of Vitamin D (1,25(OH)_2_D). PTH stimulates osteoclast differentiation and survival and calcium release from the bone matrix when serum calcium levels are low. Negative feedback avoids uncontrolled PTH-induced bone loss, by stimulating 1,25(OH)_2_D synthesis. The active form of vitamin D inhibits PTH production and stimulates intestinal calcium absorption and FGF23 expression in bone (which leads to bone phosphorus loss) (241, 243). Although, it has been described as a pre-existing condition before BS, hyperparathyroidism has been reported after gastric bypass, LAGB, SG, and BPD (265, 275, 279–281). The prevalence of hyperparathyroidism increases with the time after BS, with higher prevalence after gastric bypass followed by RYGB, LAGB, and SG (281). Nevertheless, Tsiftsis et al. failed to find differences between pre-operative and post-operative PTH levels after BPD (258) and Guglielmi et al. found a decrease in PTH levels 6 months after SG (282). Likewise, hypovitaminosis D has been widely reported in obese patients prior to surgery. This status is sustained and even worsened after BS despite post-surgery vitamin D supplementation (283). It should be mentioned that BS patients usually receive vitamin D supplementation, but there is no consensus on the dose or duration of vitamin D supplementation. This should be taken into account for inter-study comparisons as this could be limiting conclusions regarding the magnitude of the effect of each BS procedure. Some studies compared 25(OH)D levels after different bariatric procedures in which patients were given the same vitamin D dose, and no differences were found between LAGB and RYGB on vitamin D deficiency incidence (284, 285). However, higher 25(OH)D levels were found after SG than RYGB after 2 years of follow-up (276, 286).

Vilarrasa et al. could not confirm these results when the administered doses of vitamin D were higher in the RYGB than in the SG group (254). This suggests that vitamin D supplementation should be adjusted depending on the bariatric procedure as 25(OH)D might be differentially affected depending on the technique. In addition, previous vitamin D deficiency and supplementation could be affecting post-surgical hypovitaminosis D. Furthermore, despite reaching normal 25(OH)D levels after BS, a higher incidence of bone diseases was reported after BPD which suggests that mechanisms other than PTH and vitamin D regulation could be affecting post-operative bone health (257).

Taking into account the limitation mentioned above regarding bone health assessment, bone turnover markers may also be an accurate approach to verify bone resorption and formation rates after BS. N-terminal propeptide of type 1 procollagen (P1NP) (cleaved from type 1 procollagen for collagen fiber assembling when incorporated into the bone matrix), bone-specific alkaline phosphatase (BAP) (a specific marker of bone formation osteoblast activity) and osteocalcin (a bone-specific protein produced by mature osteoblasts during bone matrix synthesis) are used as bone formation biomarkers. C-terminal and N-terminal telopeptides of type I collagen (CTX-1 and NTX-1, respectively; produced as a secondary product of collagen proteolytic cleavage during bone matrix degradation by osteoclasts) and tartrate-resistant acid phosphatase (TRACP; an enzyme highly expressed by osteoclasts) are considered bone resorption biomarkers (243, 287). SG and gastric bypass led to an increase in N-telopeptide but SG was also found to increase bone alkaline phosphatase (260). BPD also increased both bone formation and bone resorption markers. Despite this, BMD was reduced after surgery (258, 275). One year after LAGB, telopeptidases were reduced and correlated with bone loss (265). RYGB also decreased CTX 1 year after surgery (246). Crawford et al. recently analyzed the effect of weight loss through intensive medical therapy or by BS (RYGB or SG) on osteocalcin and CTX at 5 years. They found that surgical patients had increased levels of both CTX and osteocalcin, but no significant effect was seen with intensive medical therapy. A more noticeable effect was seen after RYGB than in SG (288).

Although some authors have questioned the mechanical unloading hypothesis (243), molecular mechanisms have been proposed to support this. Osteocytes act as mechanostat and comprise cellular response to environmental response to bone mechanical stress. Osteocytes sense mechanical bone deformation for mechanical loading and sclerostin production is inhibited. Sclerotin negatively regulates osteoblast differentiation and function and, conversely, sclerotin is upregulated under mechanical unloading. Thus, mechanical unloading could impair bone formation by impairing osteoblast function (241). Malnutrition, particularly after mainly malabsorptive techniques, has been also suggested as being responsible for bone metabolism deficiencies such as hypovitaminosis D or hyperparathyroidism as detailed above. But, further to mechanical stress and malnutrition, other hormonal mechanisms related to the well-described hormonal changes after the various bariatric procedures have been proposed (241). GLP-1, GIP, PYY, and ghrelin have been shown to act directly in bone (45, 46, 289). GIP inhibits osteoclast differentiation and its levels have been associated with bone formation markers (290, 291). Thus, the reported GIP decrease after BS procedures implies that proximal gut bypass could be associated with negative effects on bone. On the other hand, hormonal changes dependent on procedures involving stomach fundus removal such as SG, could also impair bone formation since ghrelin directly promotes osteoblast differentiation and proliferation (292), and its levels drop after this type of surgery. The relative function of GLP and PYY on bone metabolism and changes in their levels after BS do not correlate well, presupposing a harmful effect of BS on bone. PYY deficiency has been associated with low BMD (293), but its levels increase after RYGB, LAGB, SG, and BPD. Likewise, GLP-1 levels also rise after BS procedures such as BPD, but exogenous administration of GLP-1 and GLP-2 have been shown to improve BMD (294). Adipokines that decrease after BS, such as leptin, positively affect bone metabolism (295). On the other hand, adiponectin, which has been reported to raise after BS, has been negatively correlated with BMD (296). Moreover, adipose tissue is a source of estrogens which decrease bone resorption and enhance bone formation. Therefore, a weight loss-dependent estrogen decrease could also be associated with bone metabolism impairment after BS (223, 296).

All in all, there is sufficient evidence to assume that BS has an effect on bone metabolism. However, due to the fact that multiple mechanisms affected by BS could affect bone health in opposite ways, further research is needed to elucidate the net effect of each bariatric procedure on bone metabolism. Consensus on the best way to estimate bone health, taking into account technical limitations, should also be reached to obtain clear conclusions about what is really happening to bones in obese patients before and after surgery. Long-term prospective studies evaluating fracture risk and bone disease prevalence after BS, with large sample sizes and considering the type of post-surgery vitamin D and calcium supplementation, are further required to confirm BS effects on bone metabolism and to provide patients with accurate recommendations following each bariatric procedure.

## Conclusion

Effects on metabolism and gastrointestinal hormones of BS are widely recognized, but the precise mechanisms are not yet completely understood. Moreover, it has been suggested that the different bariatric procedures are not equally effective in T2DM remission. However, heterogeneity regarding the criteria to define T2DM remission after BS makes difficult to state which surgical procedure is the most recommendable. Furthermore, other aspects than those exclusively metabolic should be taken into consideration since many other endocrine effects, but less explored up to date, are produced secondarily by the different bariatric procedures affecting corticotropic, somatotropic and gonadal axes as well as bone metabolism. Nevertheless, study design and criteria to evaluate the secondary consequences of BS are also heterogeneous which complicates the characterization of the precise effects of each bariatric procedure on these aspects. In addition, only few studies have evaluated BS consequences in the very long-term finding nutrient deficiency-related disorders, particularly after mainly malabsoprtive procedures which are at the same time considered as the most effective regarding T2DM resolution. Thus, although it is generally considered that benefits counteract by far the drawbacks of BS, further studies with homogeneous criteria and design, considering other bariatric consequences than merely metabolic effects, as well as the performance of a close long-term follow-up, are necessary in order to have a wider comprehensive overview of the consequences of each bariatric procedure. This would be useful for individualized surgical intervention recommendations depending on the different obesity co-morbidities previous to surgery of each patient as well as for preventing health-derived complications of BS.

## Author Contributions

IC-P, MC-P, and FT contributed to the manuscript design, to researching the literature and to the discussion, drafted and wrote the manuscript, and approved the final version.

### Conflict of Interest Statement

The authors declare that the research was conducted in the absence of any commercial or financial relationships that could be construed as a potential conflict of interest.
